# What Constitutes a Homœopathic Physician

**Published:** 1890-04

**Authors:** P. P. Wells

**Affiliations:** Brooklyn, N. Y.


### WHAT CONSTITUTES A HOMŒOPATHIC PHYSICIAN?

P. P. Wells, M. D., Brooklyn, N. Y.

Commissioner—*What, in your judgment, constitutes a homœopathic physician?*

Physician (a witness under examination)—*Belonging to our Society.*

It would seem that this testimony of the doctor has received a good deal of attention from many doctors, if we may judge from the comments made upon it. It is not at all surprising that it has satisfied so few. Belonging to a church does not constitute man or woman a Christian, but instead, certain qualities of head, heart, and character. It ought to surprise no one, this answer of the witness.

All are bound to accept it as the best he knew. That he knew no better should surprise no one, if we are to believe the statement of another doctor of Gotham, to the effect that after Dr. Bayard's death there was not one such physician in that city. It is not surprising if so great rarity of the fact should have begotten so great ignorance of it as to have left the poor witness with no better answer. Did not know the fact when it was seen, seems to have been what was the matter. Then how should he know what "*constituted*" the fact of which he was so evidently ignorant? If it be replied, he was before this commissioner as a witness who knew much, we can only reply, true; but this only shows more definitely the extent of his ignorance. Did not even know that he did *not* know! Then sarcasm of comment should give place to compassion.

We have said there has been much comment on this answer to the commissioner, but of those who have expressed dissatisfaction with *this* answer, not one of them has given a different one. No one has told what, in his opinion, does "*constitute*" a doctor a *homœopathic* physician. Why have they not given this? Is it because the professional strabismus which made this the best possible this witness could give has become so common that writers have, practically, only *one* eye, and that so exclusively fixed on "our Society," that they have no vision of the law of cure, or the system of therapeutics founded thereon? Is it true that this strabismus disqualifies for looking at more than one object at a time, or does it inflict on its victim total blindness as to whatever of homœopathic truth? It may be that it is sometimes the one, and sometimes the other. The probability of this is suggested by the so great absence of all relating to *homœopathic* law, philosophy, or practice, from the recorded doings of Institute, Societies, and periodicals. Indeed, judging from these, it would seem very much as if law and philosophy had become mostly obsolete ideas with self-styled homœopathists, and only "our Society" remains for our care and confidence.*

As a matter of fact, has therepeutic law ceased to exist? Has this really given place to the ever shifting, and sometimes vaunted rule of "what *I think* will do my patient good"? The second, third, and fourth men may each *think* different; then what? And in what is this shifting rule, which is really no guide, and never can be, better than the infallible, God-given law which tells what the patient should have, and never makes mistakes?

*The question*—If there be such a law, and if it is named *Homœopathy*, then it must be composed of elements which may be understood. Having its origin not only in the Maker of the law, but also in the Maker of the *mind of man*, it must be comprehensible by the powers of that mind. It must be composed

---

* To so great extent has this come to pass that at a late session, when one of its constituent members was listening to a discussion in the A. I. H., by members present, his amazement overcame his discretion so far that he cried out: "*I thought* I was in a *homœopathic* Institute!" He was not, and his disgust and disappointment were great.

of principles which together constitute the philosophy of that law. The "science of therapeutics" must be founded on those principles.

Then a *homœopathic* physician must be one who knows that law, is intelligent of its principles of philosophy, and the system of therapeutics founded on them. He must be one who believes this law, accepts its philosophy, and practices the system of therapeutics founded on it. And if he be a man of *good* conscience, a man of loyalty to law, he will at no time, and never practice aught else till he has found something more reliable and more valuable than God's law. *Such* a man *is* a "*homœopathic physician*," and *no other* man is. The man who adopts the rule of doing for the relief of the sick by means outside the law, of whatever character, exalts his petty thoughts above divine authority. He ceases by so doing, then and there, *quo ad hoc*, to be a homœopathic physician, whatever be the Society to which he belongs. He thinks himself superior to the authority of the Almighty, would seem to be the true diagnosis of his status.

